# Supplementary material for: A Nationwide Survey Investigating the Current Status of Genetic Counseling in Newborn Screening in Japan
Source: Int J Neonatal Screen. 2025 Nov 28;11(4):109. doi: 10.3390/ijns11040109 (PMC12734381; doi:10.3390/ijns11040109)
Supplement: Supplementary file 1 [file IJNS-11-00109-s001.zip › IJNS-3890398-supplementary figure S1 and table S1.pdf]

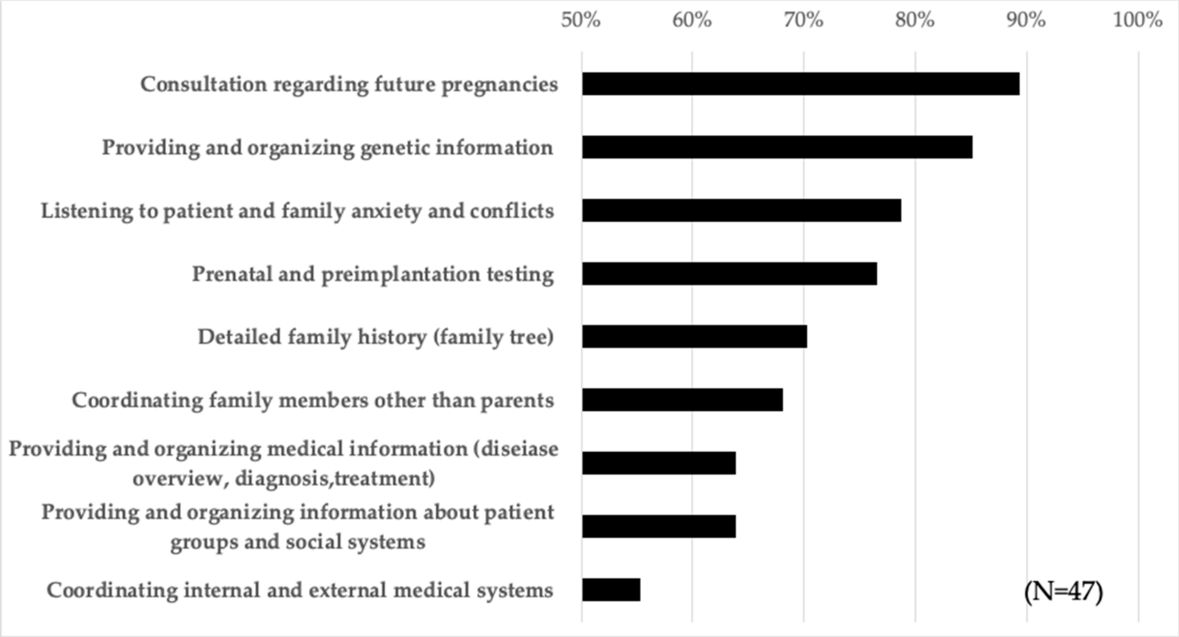

Figure S1: What metabolic specialists expect from GC for individuals who screen positive through NBS.

Table S1: Genetic care system in referral center for NBS.

Questions/Answers	n	(%)
Q1. Facility Affiliation (n=47)		
University hospitals	39	83.0%
General hospitals other than university hospitals	4	8.5%
Perinatal medical centers other than university hospitals	3	6.4%
Others	1	2.1%
Q2. Division of clinical genetics(n=47)		
Yes	41	87.2%
No	6	12.8%
Q3. Clinical geneticists(n=47)		
None	1	2.1%
1-4persons	15	31.9%
5-9persons	22	46.8%
10-14persons	5	10.6%
More than 15persons	4	8.5%
Q4. Clinical geneticists with division of clinical genetics as primary department(n=46)		
None	21	45.7%
1 person	12	26.1%
2 persons	3	6.5%
3 persons	7	15.2%
4 persons	3	6.5%
Q5. CGC (n=47)		
None	9	19.1%
1 person	18	38.3%
2 persons	11	23.4%
3 persons	5	10.6%
More than 4 persons	4	8.5%
