# Supplementary material for: A Nationwide Survey Investigating the Current Status of Genetic Counseling in Newborn Screening in Japan
Source: Int J Neonatal Screen. 2025 Nov 28;11(4):109. doi: 10.3390/ijns11040109 (PMC12734381; doi:10.3390/ijns11040109)
Supplement: Supplementary file 1 [file IJNS-11-00109-s001.zip › IJNS-3890398-supplementary file S1.pdf]

Survey on the Provision of Genetic Counseling for NBS-Positive Cases

I. Attribution

II. Implementation of genetic counseling in genetic testing for NBS-positive newborn.

III. Opinions regarding genetic counseling patients who screen positive through NBS

*This questionnaire takes approximately 10-15 minutes to complete.

Introduction

Would you be willing to participate in this survey?

□Yes □No

I. Attribution

About the Respondents

Q1. Area of expertise

□Pediatrics □Other（ ）

Q2. Qualifications of clinical geneticists

□Qualified □Not Qualified

Q3. Main department

□Pediatrics □Division of clinical genetics □Other（ ）

If you hold concurrent appointments, please specify. （ ）

Respondents' affiliated institutions

Q4. Facility Affiliation

□University hospitals

□General hospitals other than university hospitals

□Perinatal medical centers other than university hospitals

□Others

Q5. Does your facilities have a division of clinical geneticists?

□Yes □No

Q6. The number of clinical geneticists

Full-time position：（ ） persons of whom ( ) primarily assigned to the division of clinical genetics.

Part-time positions*：（ ） persons of whom ( ) primarily assigned to the division of clinical genetics.

*Examples of part-time positions: non-regular employment, short-term temporary work, affiliation with another institution as the primary workplace

Q7. The number of certified genetic counselors

Full-time position*：（　　）persons

Part-time positions：（　　）persons of whom (　　) primarily assigned to the division of clinical genetics.

*Full-time employment is defined as working five or more days per week.

Respondent's experience/knowledge of genetic counseling

Q8. Do you routinely treat genetic disorders? □Yes □No

Q9. Do you know about genetic counseling? □Yes □No

If you answered "Yes" to Q9, please respond to Q10-Q12.

Q10. Do you have a referral source if you think genetic counseling is needed?

□Yes □No

Q11. Do you have experience referring patients to genetic counseling?

□Yes □No

Q12. Do you have experience in providing genetic counseling?

□Yes □No

II. Implementation of genetic counseling in genetic testing for NBS-positive newborn.

*1: This survey asks about the provision of genetic counseling following publicly funded newborn screening (NBS) including amino acid metabolism disorders, organic acid metabolism disorders, fatty acid metabolism disorders, carbohydrate metabolism disorders, congenital hypothyroidism, and congenital adrenal hyperplasia. "Genetic testing" refers to the germline DNA analysis.

*2: Genetic counseling is defined as " the process of helping people understand and adapt to the medical, psychological and familial implications of genetic contributions to disease." In the context of this survey, "genetic counseling" refers to sessions separate from regular pediatric care, in which either a clinical geneticist or a person with training in genetic counseling provides both informational

and psychosocial support.

Q13. Do you perform genetic testing for NBS-positive newborns?

□Yes □No

If you answered “Yes” to Q13, please respond to Q14-Q17.

Q14. Genetic counseling Implementation

□Routine performance

□Conditional performance

□Not performed

Q15. If you selected “Conditional performance” in Q14, please specify the conditions (multiple answers allowed)

□Need parental support

□Need assistance in explaining the disease overview(自然歴、診断、治療)

□Interpretation of genetic test results

□Coordination of in-facility and out-of-facility care

□Future reproductive consultation

□Consideration of prenatal/preimplantation testing

□Suspicious family history or at-risk individuals

□Disease-specific considerations

□Others（ ）

If you selected “Routine performance” or “Conditional performance” in Q14, please answer the following questions Q16-Q17.

Q16. Place of implementation

□Pediatrics

□Division of clinical genetics

□Others （ ）

Q17. Genetic counseling provider (multiple answers allowed)

□Physician responsible for diagnosis and treatment of infants who screen positive through NBS

□Pediatricians distinct from those described above（clinical geneticist）

□Pediatricians distinct from those described above（non- clinical geneticist）

□Physician other than pediatricians distinct from those described above（clinical geneticist）

□Physician other than pediatricians distinct from those described above（non- clinical geneticist）

□Certified genetic counselor

□Others（　　　　　　　　）

III. Opinions regarding genetic counseling for patients who screen positive through NBS

*1: This survey asks about the provision of genetic counseling following publicly funded newborn screening (NBS) including amino acid metabolism disorders, organic acid metabolism disorders, fatty acid metabolism disorders, carbohydrate metabolism disorders, congenital hypothyroidism, and congenital adrenal hyperplasia. "Genetic testing" refers to the germline DNA analysis.

*2: Genetic counseling is defined as " the process of helping people understand and adapt to the medical, psychological and familial implications of genetic contributions to disease." In the context of this survey, "genetic counseling" refers to sessions separate from regular pediatric care, in which either a clinical geneticist or a person with training in genetic counseling provides both informational and psychosocial support.

For the following questions Q18-19, please select the response that best applies.

Q18. Genetic counseling is useful for follow-up of the child (initiation and continuation of treatment)

□ Strongly agree

□ Somewhat agree

□ Not sure / Unsure

□ Somewhat disagree

□ Strongly disagree

Q19. Genetic counseling is useful for follow-up of the parents (consideration of future pregnancies and health care)

□ Strongly agree

□ Somewhat agree

□ Not sure / Unsure

□ Somewhat disagree

□ Strongly disagree

Q20. What do you expect from genetic counseling for parents who screened positive through NBS. *Multiple choice

□Providing and organizing medical information (Disease overview, diagnosis, treatment)

□Providing and organizing genetic information

□Providing and organizing information about patient group and social systems

□Listening to anxiety and conflict

□Detailed family history (family tree)

□Consultation on future pregnancy

□Prenatal and preimplantation testing

□Coordinating family members other than parents

□Coordinating internal and external medical systems

□Unsure

□Others （ ）

Q21. Do you sometimes not provide genetic counseling to parents who screened positive through NBS?

□Yes □No

Q22. If you answered "Yes" to Q19, please indicate the reasons (multiple answers allowed).

□The problem can be solved by regular pediatric care

□Immediate treatment is the highest priority

□The child was in good condition and medical intervention was not necessary

□No person or time to provide GENETIC COUNSELING within pediatrics

□Division of clinical genetics exists at own facility, but appointments are difficult to make

□No division of clinical genetics at own facility and no referral sources

□High cost burden

□Need to coordinate on a different day (to separate from insurance care)

□I do not know what to request

□I am not sure when it should be provided

□Limited (no) needs of patients and families

□GENETIC COUNSELING is not necessary

□Others（ ）

Finally

If you have any strategies or practices you implement in genetic counseling for NBS-positive cases, please describe them. (Free text)

If you have any challenges or difficulties in genetic counseling for NBS-positive cases, please describe them. (Free text)

This concludes the survey. Thank you very much for your cooperation.
